# Hyponatremia in a Patient With Vasodilatory Shock Due to Overdose of Antihypertensive Medications: A Case Report

**DOI:** 10.7759/cureus.45053

**Published:** 2023-09-11

**Authors:** Abdul Hannan A Rasheed, Kavitha Vellanki, Frank Woo, David J Leehey

**Affiliations:** 1 Nephrology, Loyola University Medical Center, Maywood, USA; 2 Medicine/Nephrology, Edward Hines, Jr. VA Hospital, Hines, USA; 3 Medicine/Nephrology, Loyola University Medical Center, Maywood, USA; 4 Internal Medicine - Pediatrics, Loyola University Medical Center, Maywood, USA

**Keywords:** shock without endotoxemia, endotoxemia, vasopressin, vasodilatory shock, anti-hypertensive medications overdose, hyponatremia

## Abstract

Vasodilatory shock can be caused by septic shock, neurogenic shock, anaphylaxis, drugs, and toxins. Vasopressin is commonly used for the restoration of vasomotor tone in vasodilatory shock due to sepsis. This agent exerts its vasoconstrictive effect via smooth muscle V1 receptors and has antidiuretic activity via kidney V2 receptors. Stimulation of V2 receptors results in the integration of aquaporin 2 channels into the apical membrane of collecting ducts leading to free water reabsorption. This antidiuretic action of vasopressin predisposes to hyponatremia. Yet, the development of hyponatremia with the use of vasopressin in critically ill patients with sepsis is rare. A 75-year-old female presented after a suicidal attempt by ingestion of amlodipine and lisinopril. Despite adequate intravenous fluids administration, she remained hypotensive, requiring the initiation of vasopressors. She developed hyponatremia after initiation of vasopressin due to the absence of endotoxemia, and her serum sodium normalized once vasopressin was discontinued. We recommend monitoring for hyponatremia as a complication of vasopressin, especially in patients without sepsis.

## Introduction

Hyponatremia is the most common electrolyte abnormality in hospitalized and critically ill patients, with a prevalence of 15% to 30% [1]. The elderly population is at increased risk of developing hyponatremia due to the presence of various factors, including frequent prescription of drugs associated with hyponatremia, different diseases contributing to increased antidiuretic hormone, and other mechanisms, including “tea and toast” syndrome [2]. Up to 40% to 70% of cases of hyponatremia are iatrogenic/hospital-acquired [3]. Although total body salt content may be low, most hyponatremias arise from electrolyte-free water retention [4]. In septic shock, endogenous vasopressin level decreases in later stages, thus vasopressin is usually used in addition to norepinephrine for its vasopressor effects. In addition to vasopressor effects, vasopressin also has antidiuretic effects. However, hyponatremia is not frequently encountered with the use of vasopressin due to downregulation of V2 receptors in septic shock. Here, we present a rare case of iatrogenic hyponatremia with exogenous vasopressin use for the management of hypotension from an intentional overdose of antihypertensive medications.

This article was previously presented as an oral presentation at the National Kidney Foundation of Illinois (NKFI) Citywide Grand Rounds in Chicago, IL, on September 15, 2022.

## Case presentation

A 75-year-old female with a past medical history of hypertension and untreated depression presented after a suicidal attempt by ingestion of amlodipine 5 mg and lisinopril 40 mg (60 tablets each). Her blood pressure (BP) was 99/53 mmHg on presentation, pulse rate was 62/minute, respiratory rate was 14/minute, temperature was 98.9°F, and oxygen saturation was 98% on room air. Serum chemistries are shown in Table 1.

**Table 1:** Serum chemistries at presentation CO2: carbon dioxide; BUN: blood urea nitrogen; eGFR: estimated glomerular filtration rate.

Component	Result	Reference range and units
Sodium	136	136 – 144 mmol/L
Potassium	4.6	3.3 – 5.1 mmol/L
Chloride	102	98 – 108 mmol/L
CO2	16	20 – 32 mmol/L
Anion gap	18	4 – 16
BUN	41	7 – 22 mg/dL
Creatinine	2.15	0.6 – 1.4 mg/dL
Glucose	128	70 – 100 mg/dL
Calcium	10.1	8.5 – 10.5 mg/dL
eGFR	23	>89 mL/min/1.73m^2^

Of note, her serum creatinine one year before this admission was 1.0 mg/dL. Urine drug screen as well as plasma alcohol, salicylate, and acetaminophen levels were negative. She received 2 L of normal saline for hypotension along with glucagon 10 mg (5 mg x 2), and calcium gluconate 1 g as antidotes of calcium channel blocker. She continued to have hypotension despite these measures, so she was started on norepinephrine; vasopressin and epinephrine were subsequently added to maintain normal mean arterial pressures. She was noticed to have twitching in her facial muscles and right arm, for which she received levetiracetam. CT head was negative for any abnormality. Her serum creatinine improved to her baseline within 24 hours of presentation, and she was continued on norepinephrine and vasopressin for maintenance of her BP. Within 12 hours of normalization of serum creatinine, her serum sodium level decreased from 141 to 125, later reaching a nadir of 120. Serum and urine studies for workup of hyponatremia are shown in Table 2.

**Table 2:** Blood and urine tests for hyponatremia evaluation

Component	Result	Reference range and units
Serum osmolality	264	280 – 305 mOsm/kg
Serum uric acid	2.1 mg/dL	2.6 – 6.4 mg/dL
Urine osmolality	537	300 – 1000 mOsm/kg
Urine sodium	73	Variable (mmol/L)
Urine potassium	70.5	Variable (mmol/L)
Urine chloride	133	Variable (mmol/L)
Urine creatinine	66.2	Variable (mmol/L)

**Figure 1:**
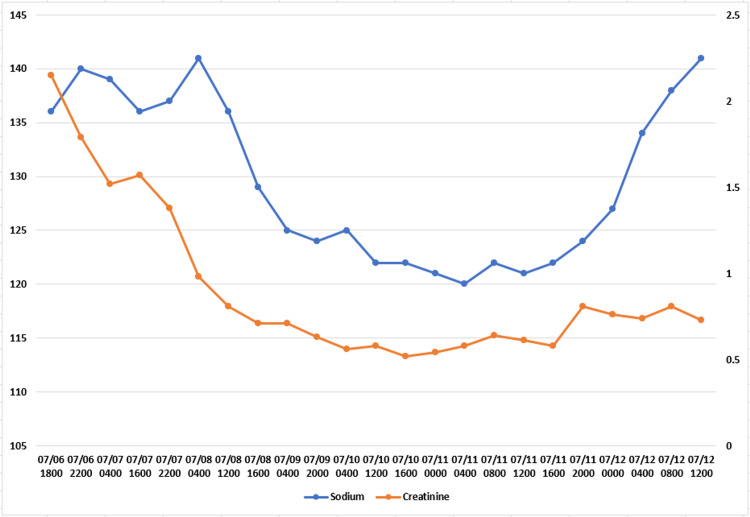
Serum sodium and serum creatinine trends during hospitalization The patient was on vasopressin from 07/07 (0505) to 07/11 (1600), on levetiracetam from 07/07 (0400) to 07/10 (2133), and on norepinephrine from 07/07 (0130) to 07/12 (1200). Drop in serum sodium and then normalization correlating with duration, the patient was on vasopressin. Creatinine in mg/dL and sodium in mmol/L.

**Figure 2:**
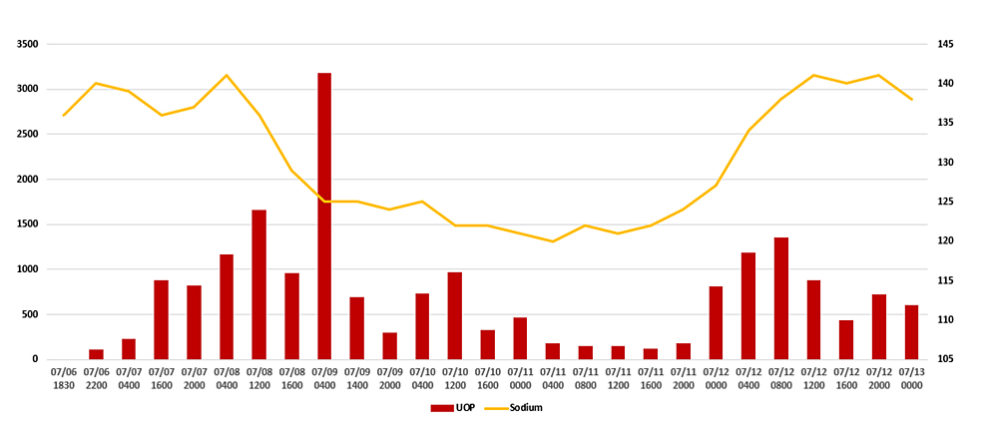
Serum sodium and urine output during hospitalization The patient was on vasopressin from 07/07 (0505) to 07/11 (1600). Urine output in mL and sodium in mmol/L.

CT head, chest X-ray, and COVID-19 were negative, ruling out central and pulmonary causes of the syndrome of inappropriate antidiuretic hormone release. Her fluid intake was restricted to 1 L/day, but her serum sodium did not improve despite fluid restriction. Levetiracetam was discontinued, without any change in serum sodium. Her BP improved and her vasopressin was tapered off on day five of admission, after which she had rapid diuresis of free water with resultant normalization of serum sodium (Figures 1, 2).

She was subsequently discharged to a psychiatric facility for continued care.

## Discussion

Amlodipine is a dihydropyridine calcium antagonist that blocks the L-type calcium channels on vascular smooth muscle, thus reducing peripheral arterial resistance and BP. It has a large volume of distribution (21 L/kg) with a high degree of protein binding (98%). In patients with normal renal function, it is slowly cleared with a terminal elimination half-life of 40 to 50 hours [5]. Calcium channel blockers (CCB) are used for the treatment of hypertension, angina pectoris, and other clinical conditions. The potential toxicity of these agents is often underappreciated. Almost 9500 cases of CCB intoxication were reported to poison centers in the United States during 2002, including intentional or unintentional overdose [6]. Dihydropyridine intoxication results in arterial vasodilation and reflex tachycardia; however, there can also be myocardial depressant effects, resulting in bradycardia. Amlodipine induces nitric oxide-dependent vasodilatation in coronary and peripheral arteries and may inhibit the angiotensin-converting enzyme (ACE) itself [7]. In conjunction with ACE inhibitors or angiotensin receptor blockers (ARBs), these complex effects might worsen toxicity [8]. Management of patients with CCB intoxication depends on the severity of symptoms, with interventions including intravenous crystalloids, atropine, calcium salts, glucagon, high-dose insulin/glucose infusion, vasopressors, and intravenous lipid emulsion therapy [9,10]. Orogastric lavage is effective only in patients who present within one to two hours of ingestion.

Vasodilatory shock is characterized by a failure of peripheral vasoconstriction in the face of low systemic arterial pressure [11], which, in our case, was caused by intentional intoxication of anti-hypertensive medications. In addition to volume resuscitation, vasopressors are usually required for the management of vasodilatory shock. Norepinephrine is the first-line agent for its potent vasoconstrictive effects as well as a modest increase in cardiac output [12]. Vasopressin is a second-line agent in refractory vasodilatory shock to improve BP and reduce the dose of the first-line agent. Vasopressin is both a vasopressor and an antidiuretic hormone. It has vasoconstrictive effects via V1 receptors as well as antidiuretic effects via V2 receptors on collecting ducts [13]. A fall in endogenous vasopressin levels may occur in the late stages of shock; exogenous vasopressin is thus frequently utilized in refractory shock [14]. Although plasma hypertonicity serves as the primary stimulus for arginine vasopressin (AVP) release under normal conditions, the responsiveness of the osmoreceptor mechanisms appears to be significantly altered by modest changes in blood volume, indicating a close interrelationship between the two variables in the control of AVP release [15]. The potential consequence of exogenous vasopressin administration is water intoxication with subsequent hyponatremia. Despite the common use of vasopressin in intensive care settings (the median hospital rate of vasopressin use for septic shock was 11.7 [16]), hyponatremia is a rare complication of vasopressin. In a randomized double-blind, vasopressin and septic shock trial (VASST), vasopressin was compared with norepinephrine in septic shock patients (382 patients randomized to receive norepinephrine and 396 patients randomized to receive vasopressin), only one patient developed hyponatremia (defined as <130 mEq/L in this trial) in each group (0.3%) [17]. This can be explained by downregulation of vasopressin V2 receptors in septic shock. This has been shown in the peritoneal endotoxin-challenged rat model, where lipopolysaccharide (LPS) injection was associated with a decrease in V2 vasopressin receptors as well as a decrease in aquaporin 2 in the kidney [18]. Coadministration of corticosteroids and catecholamines with vasopressin also reduces the risk of development of hyponatremia in septic shock patients [19].

We summarize that the absence of endotoxemia in our patient resulted in the development of hyponatremia. This phenomenon has been shown in experimental models in the past as well, where the administration of vasopressin (Pitressin) and water to normal subjects resulted in the development of hyponatremia [20]. Another possible predisposing factor in our case was the presence of normal renal function at the time of its occurrence, an uncommon finding in septic patients. After the improvement of BP, discontinuation of vasopressin resulted in rapid diuresis and normalization of serum sodium levels in our patient without any additional interventions.

## Conclusions

Vasopressin is one of the most commonly used vasopressors in patients with septic shock. Despite the antidiuretic effect of vasopressin, the development of hyponatremia is rare in septic patients due to endotoxemia-mediated downregulation of V2 receptors and likely resistance to its antidiuretic effects due to concomitant acute kidney injury (AKI). In our case, we hypothesize that the absence of endotoxemia and resolution of AKI led to free water retention and hyponatremia with vasopressin use and rapid free water excretion and normalization of serum sodium with cessation of vasopressin. This case highlights the importance of this rare but potentially serious side effect of vasopressin.
